# Alveolar Soft Part Sarcoma: A Single-Center 26-Patient Case Series and Review of the Literature

**DOI:** 10.1155/2012/907179

**Published:** 2012-05-15

**Authors:** Koichi Ogura, Yasuo Beppu, Hirokazu Chuman, Akihiko Yoshida, Noboru Yamamoto, Minako Sumi, Hirotaka Kawano, Akira Kawai

**Affiliations:** ^1^Division of Musculoskeletal Oncology, National Cancer Center Hospital, 5-1-1 Tsukiji, Chuo-ku, Tokyo 104-0045, Japan; ^2^Department of Orthopaedic Surgery, The University of Tokyo Hospital, 7-3-1 Hongo, Bunkyo-ku, Tokyo 113-8655, Japan; ^3^Division of Pathology, National Cancer Center Hospital, 5-1-1 Tsukiji, Chuo-ku, Tokyo 104-0045, Japan; ^4^Division of Medical Oncology, National Cancer Center Hospital, 5-1-1 Tsukiji, Chuo-ku, Tokyo 104-0045, Japan; ^5^Division of Radiation Oncology, National Cancer Center Hospital, 5-1-1 Tsukiji, Chuo-ku, Tokyo 104-0045, Japan

## Abstract

*Background.* Alveolar soft part sarcoma (ASPS) is a rare tumor, and little information is available regarding its clinical features and appropriate treatments. *Methods.* A retrospective review of 26 consecutive ASPS patients (12 male, 14 female; mean age of 27 years) treated at our institution over 30 years (mean followup; 71 months) was performed. *Results.* The primary tumor developed in the lower extremity (12), trunk (8), and upper extremity (6), with an average size of 7.2 cm (range, 2–14 cm). The AJCC stage at presentation was IIA (7), III (3), and IV (16). Surgical excision was performed in 20 patients (R0 18, R1 plus radiotherapy 2) without local recurrence. Six patients (stage IIA 3/7, stage III 3/3) later developed metastases after an average period of 28.7 months. The median survival of the 26 patients was 90 months, with overall 5/10-year survival rates of 64%/48%. AJCC stage and tumor size were significant prognostic factors. Significant palliation and slowing of metastasis progression were achieved with gamma knife radiotherapy. Nine patients receiving chemotherapy showed no objective response. *Conclusions.* ASPS is indolent but has a high propensity for metastasis. Early diagnosis and complete excision of the small primary tumor are essential in the treatment of ASPS.

## 1. Introduction

Alveolar soft part sarcoma (ASPS) is a rare tumor that was initially described as a distinctive clinical entity by Christopherson et al. in 1952 [1]. It accounts for approximately 0.5–1% of all soft tissue sarcomas and affects mainly adolescents and young adults [2]. The name “alveolar” was derived from its pseudoalveolar appearance with clustered polygonal cells lacking central cohesion. ASPS tumor cells exhibit characteristic PAS-positive, diastase-resistant, intracytoplasmic rhomboid crystals that contain monocarboxylate transporter 1 and CD147 [3]. Molecular cytogenic studies of ASPS have demonstrated the chromosome rearrangement der(17)t(X;17)(p11;q25) resulting in the *ASPL-TFE3* fusion gene, which is highly specific and critical for development of the tumor [4]. In spite of these advances in basic research, the origin/differentiation of ASPS still remains obscure, and no optimal or effective treatment has been devised, especially for advanced cases.

The main obstacle to gaining a thorough understanding of the clinical behavior and optimal treatment of ASPS is the rarity of the disease. The majority of previous reports have been in the form of small collective series from referral centers or multi-institutional studies over a long period (Table 1) [5–15]. Most series have suggested that ASPS is resistant to conventional cytotoxic chemotherapy and support the contention that complete excision of the tumor is the only meaningful treatment for ASPS. Moreover, data regarding the clinical value of radiotherapy for treatment of ASPS are still limited [5–13].

With this background in mind, we conducted the present study of 26 consecutive patients with ASPS treated at our institution during the era of modern multidisciplinary treatment with a view to seeking a new or ideal form of treatment for the disease.

## 2. Patients and Methods

Between 1979 and 2008, 26 consecutive patients with ASPS were treated at our institution. Their clinical data, histopathological findings, treatment modalities, and treatment outcome were reviewed retrospectively. The followup period ranged from 8 to 311 months with an average of 71 months.

The patients underwent multimodality treatment including surgery, radiotherapy, and chemotherapy depending on the individual tumor stages. We attempted wide excision of the primary tumor to achieve a negative surgical margin whenever possible. The surgical margin was examined histologically at the point closest to the resected specimen, and was classified as R0 (negative, no tumor cells on the inked margin) or R1 (positive, tumor cells on the inked margin). Radiotherapy was used in patients who were at high risk of local recurrence after R1 excision (adjuvant radiotherapy) or in patients who had advanced or metastatic disease (palliative radiotherapy). Chemotherapy was administered to patients with metastatic disease using a variety of regimens (mainly anthracycline-containing regimens). Tumor response was judged from findings of plain radiography, computed tomography (CT), and magnetic resonance imaging (MRI) according to the Response Evaluation Criteria in Solid Tumors (RECIST) [16].

 The following demographic and treatment factors were examined for prognostic importance: patient age (≤30 years or >30 years, ≤18 years or >18 years), gender, tumor size (≤5 cm or >5 cm), tumor location and depth, surgical margin, and American Joint Committee on Cancer (AJCC) staging [17]. Tumor depth was classified as superficial or deep in relation to the investing fascia.

Continuous data were summarized as means or medians unless noted otherwise, while discrete (or categorical) data were summarized as counts (percentages). Overall, local recurrence-free and distant metastasis-free survivals were calculated using the Kaplan-Meier method. Univariate analysis was performed by log rank analysis. Differences at *P* < 0.05 were considered significant. Statistical analyses were performed using the Statview 5.0 statistical package (SAS Institute Inc., Cary, NC). This study was approved by the institutional review board of National Cancer Center.

## 3. Results

### 3.1. Clinical Characteristics

The study group comprised 12 men (mean age of 30 years) and 14 women (mean age of 24 years) with an average age at diagnosis of 27 years (range, 2–46 years). There was a slight female preponderance in our series, and this tendency was more striking in the patients aged ≤30 years (4 males and 10 females). On the other hand, there was male preponderance among the patients aged >30 years (8 males and 4 females). The primary tumors were located in the lower extremity (*n* = 12), trunk (*n* = 8), and upper extremity (*n* = 6) and averaged 7.2 cm (range, 4–14 cm) in largest diameter. Twenty five tumors were deep-seated and one was superficial. Most patients (*n* = 23) presented with a painless mass, two with asymptomatic lung metastases found at a periodic medical examination, and one with painful rib metastases. Ten patients (38%) presented with localized disease and 16 (62%) with metastatic disease: lung in 15, bone in 3, and brain in 2. According to the AJCC staging [17], 7 patients were classified as stage IIA, 3 as stage III, and 16 as stage IV.

### 3.2. Treatment

Surgical excision of the primary tumor was performed in 20 patients. The surgical margin was classified as R0 in 18 and R1 in 2. The two patients with an R1 margin received adjuvant radiotherapy (preoperative radiotherapy (46.2 Gy) in one, and postoperative brachytherapy (36 Gy) in the other) [18]. No local recurrence was seen in these 20 patients during a mean followup period of 81 months.

Two patients with stage IV disease underwent only biopsy of the primary tumor and showed slow but gradual progression of the primary tumor during followup. Four patients with stage IV disease received radiotherapy (40–66 Gy) as the only treatment for the primary tumor because of the advanced nature of their disease. None of these 4 patients showed progression of the irradiated primary tumor until the time of death or last followup (13–24 months; mean 20.5 months).

Chemotherapy in various combinations (including doxorubicin, ifosfamide, cyclophosphamide, cisplatin, etoposide, gemcitabine, and docetaxel) was administered to 9 patients with stage IV disease. No objective clinical response was observed (SD in 2, PD in 6 patients) in all of the 8 patients with measurable disease.

Five patients with localized disease ultimately developed metastases. Altogether, 21 (81%) of the 26 patients had metastatic disease, either at presentation or developing later. The sites of metastases included the lung in 20 patients, brain in 11, bone in 7, liver in 3, and spleen in 2.

### 3.3. Survival

**Figure 1:**
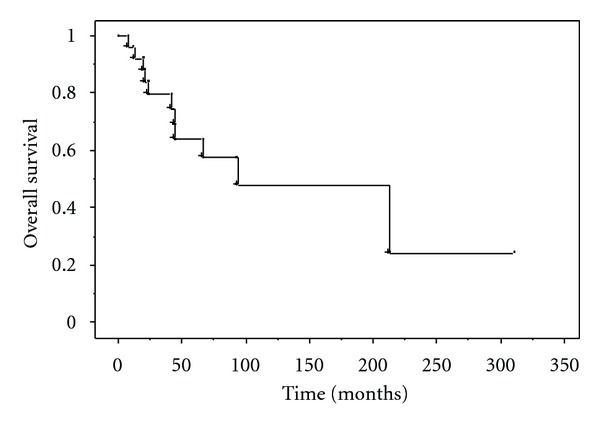
Kaplan-Meier plots showing overall survival of the 26 patients with ASPS.

**Figure 2:**
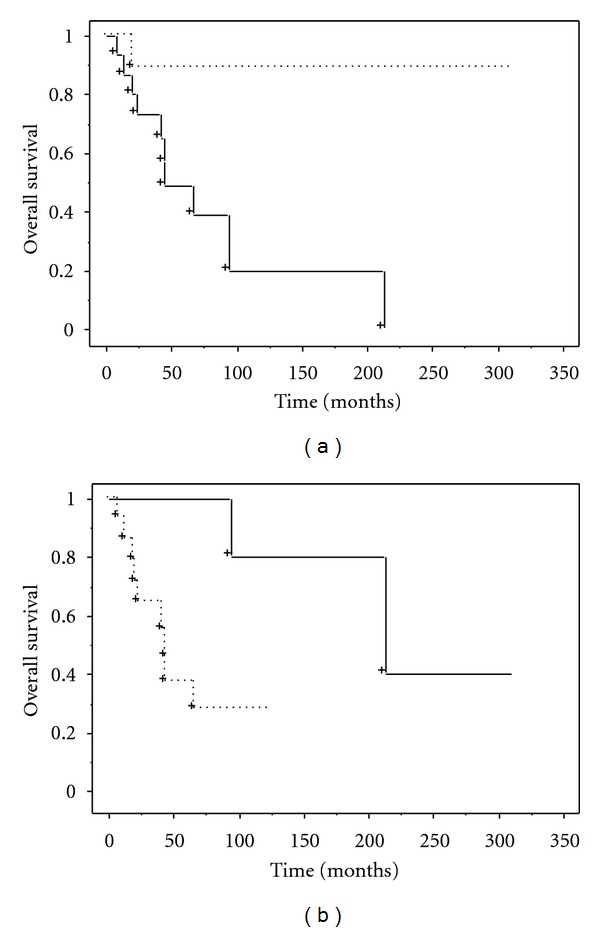
Kaplan-Meier plots showing overall survival stratified by (a) tumor size (>5 cm: solid line, ≤5 cm: dashed line) and (b) AJCC stage (stage IIA, III: solid line, stage IV: dashed line).

The median survival of the 26 patients was 90 months, with overall 5- and 10-year survival rates of 64% and 48%, respectively (Figure 1). Median survival for those with stage IV disease was 41 months. The 5-year local recurrence-free, metastasis-free, and overall survival rates for patients with localized disease at presentation were 100%, 33%, and 100%, respectively. Univariate analysis showed that tumor size (*P* = 0.014) and AJCC stage (*P* = 0.005) were significant prognostic factors for overall survival (Figure 2, Table 2).

## 4. Discussion

ASPS is an extremely rare tumor that accounts for approximately 0.5–1% of all soft tissue sarcomas [2]. The rarity of the tumor makes it difficult to draw definitive conclusions regarding its clinical characteristics, prognostic factors, and appropriate treatment. The majority of previous reports have been in the form of case reports or small collective series [5–15]. The present study is one of the largest series based on data from a single referral center in the era of modern multidisciplinary treatment [9, 12, 13].

The mean patient age at diagnosis was 27 years, and a slight female preponderance (54%) was observed overall. Interestingly, among the 14 patients aged ≤30 years, there was a striking female preponderance (10 female patients, 71%), in contrast to a male preponderance among the 12 patients aged >30 years (4 female patients, 33%). Such an age-related gender ratio inversion was also described in two previous series from the Memorial Sloan-Kettering Cancer Center and MD Anderson Cancer Center [9, 12]. As this phenomenon appears to be exceptional in the field of sarcoma/cancer epidemiology, we retrieved data from the nationwide bone and soft tissue tumor (BSTT) registry in Japan [19]. In the prospectively collected BSTT database, we found 67 patients with ASPS (30 males (45%) and 37 females (55%)) diagnosed during the period of 2002 to 2009. The same age-related gender ratio inversion was found in this nationwide cohort. Among the 40 patients aged ≤30 years, there was a definite female preponderance (27 females, 68%), in contrast to a male preponderance among the 27 patients aged >30 years (10 females, 37%).

ASPS has a nonreciprocal chromosomal translocation der(17)t(X;17)(p11;q25) with the corresponding oncogenic fusion gene, *ASPL-TFE3* [20]. Because females have an extra X-chromosome, their likelihood of developing an X-autosome translocation is theoretically double that of males. Recently, Bu and Berstein have proposed that the female preponderance in ASPS may be a result of having two X-chromosomes with a fusion gene that is not subject to X-inactivation. Although a mathematical model based on population-based SEER registry data has supported their hypothesis in ASPS as a whole, it alone is unable to account for the observed age-related gender ratio inversion. It seems that additional and/or alternative mechanisms such as hormonal effects might be involved in this intriguing phenomenon.

There is little information available in the literature regarding prognostic factors in ASPS (Table 1) [5–15]. In our series, tumor size and AJCC stage were significant prognostic factors for overall survival of patients with ASPS. The impact of tumor size on the prognosis of ASPS has also been reported in previous studies [7, 10, 13].

An effect of tumor size on the outcome of soft tissue sarcoma has been extensively demonstrated. In general, in soft tissue sarcomas, tumor size indirectly reflects the rapidity of tumor growth and tumor biological aggressiveness. However, considering the indolent tumor growth of ASPS, this concept does not appear to be applicable. In ASPS, a larger tumor might reflect a longer disease history, and thus a higher likelihood of systemic spread. Indeed, in our series, there was a significant correlation between tumor size and presence of metastases; 3 of 10 patients (30%) with tumors ≤5 cm in largest diameter presented with localized disease (AJCC stage IIA or III), whereas 12 of 15 patients (80%) with tumors measuring >5 cm presented with metastatic disease (AJCC stage IV) (*P* = 0.001, chi-squared test).

Although statistically not significant, younger age (≤18 years) at diagnosis was associated with a favorable outcome in our series. Some previous reports have also indicated that pediatric ASPS has a more favorable prognosis than its adult counterpart [9, 11]. Differences in biological characteristics between pediatric and adult ASPS have been proposed to explain the better prognosis of pediatric ASPS [7, 9]. In ASPS, a larger tumor size, as described above, is a prognostic factor that impacts on survival. In our series, the tumor at presentation showed a tendency to be smaller in pediatric patients (age ≤18 years) than in adult patients (age >18 years); only 1 of 6 pediatric patients (17%) presented with a tumor measuring >5 cm, while 14 of 19 adult patients (74%) did so (*P* = 0.001, chi-squared test). Our data indicate that the age-related difference in prognosis may be attributable to a larger tumor size, resulting in an increased risk of distant metastases in adult patients.

Local recurrence after excision of ASPS was reportedly as high as 20% in the largest series of 91 patients treated at the Memorial Sloan-Kettering Cancer Center [9]. Evans noted a higher local recurrence rate of 31% [14]. In contrast, Portera et al. and van Ruth et al. reported that the local recurrence rate of ASPS after surgical excision was around 10% [12, 15]. The risk factors for local recurrence in ASPS have not been clarified. Ogose et al. reported that none of 38 patients who underwent wide excision of ASPS developed local recurrence, in contrast to 4 out of 7 patients who underwent marginal excision without radiotherapy [10]. In our series, no local recurrence was observed in 18 patients who underwent tumor excision with an R0 margin. These results suggest that R0 excision is essential (and perhaps sufficient) for achieving satisfactory local control of ASPS.

None of the patients who underwent R1 excision plus adjuvant radiotherapy developed local recurrence in the present series. Although it is impossible to draw definitive conclusions about the effectiveness of adjuvant radiotherapy for ASPS based on the limited number of patients in our series, several studies have suggested that adjuvant radiotherapy is beneficial in this setting. Sherman et al. reported prolonged (24–150 months) local control in all of six patients who underwent surgery and adjuvant radiotherapy (56–65 Gy) [21]. Anderson et al. also reported good local control (no local recurrence) of the primary tumor in 14 patients with ASPS after wide excision and adjuvant radiotherapy (45–72 Gy) [5]. Moreover, some of their patients showed no progression of the primary tumor after radiotherapy alone without surgery. The benefit of radiotherapy for patients with ASPS appears attributable to enhanced local control after surgery with inadequate or close resection margins.

ASPS shows a high incidence (around 30%) of brain metastases, being at least 3 times higher than that of other soft tissue sarcomas [22]. Although it remains unclear whether this high incidence of brain metastases is attributable to disease-specific biology or a long disease duration, the survival of patients after diagnosis of brain metastases remains poor (median survival 12 months). As almost all brain metastases have been associated with metastatic spread to extracranial sites such as the lung, surgical treatment is not usually indicated. In our series, four patients with brain metastases underwent gamma knife radiotherapy and achieved satisfactory local control of the disease, with a median progression-free time of 12 months (range, 9–30 months). It is striking that all 4 patients were alive without local disease progression at the time of last followup. Although larger prospective studies are important for clarifying specific types of patients who would benefit from this type of radiotherapy, it is expected to become the treatment of choice for those with brain metastases from ASPS.

In the present series, none of the patients responded to, or benefited significantly from, conventional chemotherapies including a combination of gemcitabine and docetaxel. These results are concordant with the widely accepted concept that conventional cytotoxic agents such as doxorubicin, ifosfamide, and dacarbazine have little efficacy in ASPS [5–12]. Reports of tumor responses to interferon alpha (IFN-*α*) have been anecdotal [23–25]. It is apparent that alternative therapeutic strategies/agents are necessary for patients with advanced ASPS.

The feasibility of using an antiangiogenic agent for the treatment of ASPS was demonstrated by Vistica et al. using *in vivo* preclinical models [26]. They observed upregulation of angiogenesis-related genes in an ASPS xenograft model and demonstrated that a combination of bevacizumab (a humanized antiVEGF*α* monoclonal antibody) and topotecan (a topoisomerase 1 inhibitor with antiangiogenic properties) slowed the growth of the tumor by 70%. Recently, it has been reported that several antiangiogenic agents such as bevacizumab and sunitinib malate exert tumor-suppressive effects in ASPS [26–30].

Notably, we found that cediranib (AZD2171), a potent oral inhibitor of VEGFR tyrosine kinases that blocks the elaboration of new blood vessels, elicited significant tumor shrinkage with a progression-free survival period of 27 months (data not shown). Cediranib has been identified as having substantial single-agent antitumor activity against ASPS in early-phase clinical trials that included 7 patients [30]. Another recent larger phase II study has demonstrated preliminarily that cediranib has promising activity and safety with a response rate of >40% and a disease control rate (PR+SD) at 6 months of 78% [28]. Given these data, an antiangiogenic approach seems to be promising and may become a breakthrough treatment for management of advanced ASPS.

In conclusion, ASPS is relatively indolent but has a high propensity for metastasis. Early diagnosis and complete excision of a small primary tumor is important in the treatment of ASPS. Antiangiogenic strategies may become a breakthrough form of management for advanced ASPS. We propose that patients with metastatic ASPS should be enrolled in prospective clinical trials to assess the effectiveness of new treatments such as antiangiogenic therapy.

## Figures and Tables

**Table 1:** Clinicopathologic studies of ASPS.

Reference	Year	No. of patients	5-year survival (%)			Prognostic factor
			All	M0	M1	
Evans [14]	1985	13	60	NA	NA	Size
Auerbach and Brooks [6]	1987	20	67	NA	NA	NA
Lieberman et al. [9]	1989	91	57	60	22	Age, AJCC stage
Pappo et al. [11]	1996	11*	88	NA	NA	None
Casanova et al. [7]	2000	19*	80	91	NA	Size
Portera et al. [12]	2001	70	47	88	20	AJCC stage
Van Ruth et al. [15]	2002	15	38	48	NA	NA
Ogose et al. [10]	2003	57**	56	81	46	AJCC stage, size, bone involvement
Anderson et al. [5]	2005	15	75	NA	NA	None
Daigeler et al. [8]	2008	11	88	88	—	None
Pennacchioli et al. [13]	2010	33	69	NA	NA	Size, surgical margin
Current study		26	64	100	37	AJCC stage, size

NA indicates not available.

*All patients are pediatric patients.

**Patients from 27 institutions.

**Table 2:** Potential risk factors for overall survival.

Factor	No. of patients	5-year survival	*P*-value
Gender			
Male	12	80	0.57
Female	14	50	
Age at diagnosis			
≤30 years	14	66	0.21
>30 years	12	61	
≤18 years	7	83	0.11
>18 years	19	57	
Tumor size			
≤5 cm	10	89	0.014
>5 cm	15	53	
Location 1			
Upper extremity	6	83	0.31
Lower extremity	12	58	
Trunk	8	69	
Depth			
Deep	25	62	NA
Superficial	1	100	
The AJCC stage			
IIA/III	10	100	0.005
IV	16	38	
Surgical margin			
R0	18	63	0.91
R1	2	50	
